# The impact of paramedic shift work on the family system: a literature review

**DOI:** 10.29045/14784726.2019.03.3.4.43

**Published:** 2019-03-01

**Authors:** Lucy Anderson

**Affiliations:** South East Coast Ambulance Service NHS Foundation Trust

## Abstract

**Aims:**

More paramedics than ever are taking time off or leaving the ambulance service through stress; career decisions could be greatly influenced by the perceived impact of shift work on families. Through three key themes of physical injury, emotional labour and work–family fit, this review explores how paramedic shift work impacts the family system and how this could influence retention and recruitment of staff.

**Methods:**

A systematic literature search was conducted using key terms and Boolean operators, and strict inclusion and exclusion criteria. Twenty-two papers were deemed to be relevant. These were critiqued using a framework addressing both quantitative and qualitative research for the novice researcher in healthcare. In turn each theme was reviewed and discussed.

**Results:**

Due to the variety of roles required of a paramedic combined with the unpredictable environments in which they work, paramedics are at high risk of sustaining a work-related injury. Families raise concerns about paramedics’ safety at work, and the financial and psychological implications of sustaining an injury concern paramedics themselves. Additionally, links have been found between paramedic shift length and risk of occupational injury.

Paramedics rely heavily on families for emotional support owing to the nature of incidents attended at work. The male coping culture ingrained into paramedic practice deters this at work. As family members are often uneducated in emotional processing techniques, this puts them at risk of vicarious trauma. Additionally, techniques employed by paramedics to cope with emotional distress are carried into the home environment to its detriment.

Several shift characteristics (length, pattern, weekend work, high weekly work hours, inflexible schedules and low job satisfaction) contribute to work–family conflict, relationship problems, child rearing conflict and difficulties in maintaining a social life.

**Conclusion:**

Changes are required in the organisational culture, from one which denigrates staff for reporting injuries, for showing emotions and for struggling to balance their home life with work, to one which improves paramedics’ experience at work and therefore their home life too. Investment in education programmes for families on how to enable emotional processing and the risks this carries will improve the picture for families. Flexible work arrangement is an area of organisational reform that could greatly improve job satisfaction, staff retention and recruitment, and ultimately improve family life.

Further research into the areas of paramedic shift work that impact the home system, especially in the UK, is needed to better understand some of the issues with staff retention and recruitment.

